# Chemokines play a role in nerve damage and neuroprotection in vascular dementia

**DOI:** 10.1016/j.ibneur.2024.08.002

**Published:** 2024-08-05

**Authors:** Jinming Ma, Manqing Zhang, Peijie Fu, Xiaoping Yin, Zhiying Chen

**Affiliations:** a Department of Neurology, Affiliated Hospital of Jiujiang University, Jiujiang,Jiangxi, 332000, China; b Jiujiang Clinical Precision Medicine Research Center, Jiujiang, Jiangxi,332000, China; c School of Basic Medicine, Jiujiang University, Jiujiang, Jiangxi, 332000, China

**Keywords:** Chemokines, vascular dementia, nerve damage, inflammatory response

## Abstract

Various Chemotactic Factors (FCs) play different roles in neuronal injury in vascular dementia. CXCL5 and CCL11 exacerbate neurological injury by promoting inflammatory responses. CXCL12/SDF-1 and CX3CL1 play neuroprotective roles.CXCL13, XCL-1 and CCL2/ MCP-1 exacerbate neurological injury in the early stage, while exerting neuronal regeneration and neuroprotective effects in the chronic progressive phase. Chemokines often play an important role in the course of vascular dementia by regulating inflammatory responses, oxidative stress, and autophagy. Activation of microglia plays an important role in the regression of vascular dementia. Activated microglia M1 causes neuronal damage through the release of chemokines. And microglia M2 has anti-inflammatory effects and is involved in the repair of brain damage. Therefore, dynamic monitoring of various related FCs and understanding the relationship between FCs and microglia can help to understand and regulate the disease course progression of vascular dementia.At present, many scholars have confirmed in basic research that different subgroups of chemokines are closely related to vascular dementia. In clinical research, new immunotherapy methods that upregulate XCL-1 and drugs that regulate the activity of CCL2/CCR2 signaling pathways are being studied and promoted.

## Introduction

Vascular dementia (VaD) is a syndrome of cognitive dysfunction secondary to cerebrovascular disease and its risk factors, with a high incidence and insidious onset. The prevalence of VaD in China is about 1.1–3 %, and it is the second most common type of dementia after Alzheimer's disease. Its common causes include cerebral infarction, cerebral ischemia-reperfusion injury, chronic cerebral hypoperfusion (CCH), and cerebral hemorrhage. Chronic Cerebral Hypoperfusion (CCH) is a key determinant of its pathogenesis, which can cause ischemic damage to brain tissue and lead to cognitive dysfunction in the body (Rajeev et al., 2022, Zhou et al., 2022). Cerebral ischemia-reperfusion injury (CI/R) evolves over time and space, leading to progressive neurological damage, and plays a key role in the acute progressive phase of the chronic course of VaD. It has been shown to prevent cognitive impairment and blood-brain barrier disruption caused by CCH deficiency by blocking RNA-binding proteins and chemokines (Lee et al., 2022). Chemotactic Factors (FCs) are a class of small cytokines or signaling proteins that induce targeted chemotaxis of peripheral responding cells and play an important role in the pathophysiology of VaD neurological injury. Based on the number and arrangement of cysteines conserved in the N-terminal of chemokines, FCs can be classified into four subclasses, CXC, CC, C and CX3C (C is a cysteine and X is an arbitrary amino acid). It has been found that CCH can induce cognitive dysfunction through MCP-1-mediated microglia activation (Hui Shi and Changlong Zhou, 2016). xCL-1 plays an important role in neuropathy (Zychowska et al., 2016). Moreover, CXCL5, CXCL12, CXCL13, CX3CL1/CX3CR1 signaling pathway, CCL11, and SDF-1a/CXCR4 axis are significantly associated with post-ischemic neurological injury (Stanne et al., 2022, Schutt et al., 2012, Zhang et al., 2022, Meena et al., 2021, Cui et al., 2022a, Huang et al., 2019). Therefore, active knowledge of FC levels can dynamically observe the progression of VaD, which is important for effective prevention and intervention of VaD. This article summarizes the roles of different subgroups of chemokines in vascular dementia and summarizes and proposes possible clinical treatment strategies.This article aims to discuss the role of different chemokines in vascular dementia and summarize the existing clinical research.

## Overview of FCs

FCs are a class of low molecular weight proteins that attract leukocytes to migrate to the site of infection, and their main role is to manage leukocyte migration to various locations during inflammation and homeostasis in vivo, and to activate cells to initiate immune responses or promote wound healing. This article will focus on FCs closely related to VaD, including CXCL5, CXCL12/SDF-1, CXCL13, XCL-1, CX3CL1, CCL2/MCP-1 and CCL 11.

### Physiological effects of FCs

It has been shown that CXCL5 functions as a promoter of angiogenesis, CXCL12/SDF-1 promotes hematopoietic cell migration to the bone marrow and large vessel formation, and CXCL13 activates astrocytes, thereby regulating neuropathic pain.XCL-1 can be expressed by both neurons and microglia, and CX3CL1 is thought to be the origin of follicular helper T cell germinal centers. High expression of CCL2/MCP-1 in brain interneurons may be involved in maintaining emotional states. CCL11 is associated with aging.FCs can be expressed by various CNS cells and have protective or degenerative effects on CNS cells (Dhaiban et al., 2020).

### Role of FCs in the pathological environment

CXCL5 is known as a classical pro-inflammatory cytokine involved in tissue remodeling (Hummitzsch et al., 2021).CXCL12 enhances platelet aggregation (Leberzammer et al., 2022).CXCL13 reduces the recruitment of TFH cells at the site of injury and has a protective effect (Rayasam et al., 2022a).XCL-1 mediates antigen cross-presentation and activates CD4 and CD8 T cells (Xiang et al., 2021). Inflammatory cytokines such as TNF-α and IFN-γ induce CX3CL1 expression, which in turn regulates the recruitment of antigen-presenting cells (Nagata et al., 2022).CCL2 is one of the strongest signals for monocyte aggregation to sites of inflammation (Georgakis et al., 2022).CCL11 is a pro-inflammatory cytokine that is widely involved in leukocyte migration and activation (Cui et al., 2022b).

## Relationship between FCs and VaD

FCs act at different times in the course of VaD disease. Early in the course of the disease, CXCL5 mediates the generation of an inflammatory environment and CCL11 causes damage to nerves. CXCL12/SDF-1 and CX3CL1 subsequently promote nerve repair during the CI/R and CCH phases of VaD. changes in CXCL13, XCL-1 and CCL2/MCP-1 levels are critical for the advancement of the VaD disease course.Next, this article will elaborate on the chronological order of the effects of FCs and present them in Table 1 for a faster understanding.

**Table 1:** Variation and functional relationships of various FCs in VaD.

Type of FCs	Change Direction	Route of action	Research Sources
CXCL5	Upward adjustment	Promotes inflammatory response and aggravates nerve damage	Patients with cerebral ischemia and mouse models (Yu et al., 2021a, Giambrone et al., 2019, Wang et al., 2016a, Zaremba et al., 2006)
CXCL12/SDF−1	Upward adjustment	Promotes nerve cell repair and regeneration	Patients with cerebral ischemia and mouse/rat models (Wang et al., 2022a, Huo et al., 2022a, Zhao et al., 2022)
XCL−1	Upward adjustment	Specific chemotactic activity	Patients with cerebral ischemia (Habiyaremye et al., 2017)
CX3CL1	Upward adjustment	Reduction of neural cell apoptosis	Patients with cerebral ischemia and rodents (Subbarayan et al., 2022a, Bai et al., 2021a, Pawelec et al., 2020)
CCL2/MCP−1	Upward adjustment	Has neurotoxic and neuroprotective effects	Patients with cerebral ischemia and mouse models (Huang et al., 2018, Shan et al., 2021, Bernstein and Rom, 2020, Geng et al., 2022)
CCL−11	Upward adjustment	Can lead to neurodegeneration	Patients with cerebral ischemia and mouse models (Roy-O'Reilly et al., 2017, Wang et al., 2017a, Lieschke et al., 2019)
CXCL13	Upward adjustment	Promotes neuroblast migration and participates in neuroprotection	Mouse/rat model of cerebral ischemia (Rayasam et al., 2022a, Guo et al., 2020a, Chen et al., 2020a, Chapman et al., 2015, Monson et al., 2014)

FCs：Chemokines

### CXCL5 and CCL11 exacerbate nerve damage in VaD

The biological effects of CXCL5 present in brain inflammation and neurodegenerative diseases are achieved by interacting with CXCR2 receptors and activating the p38 MAPK kinase signaling pathway (Yu et al., 2021a). CXCL5 recruits neutrophils to sites of acute inflammation and has significant immunomodulatory functions (Compton et al., 2021).CCL11 has been identified as a contributing factor to neurodegeneration (Roy-O'Reilly et al., 2017) CCL11 can promote eosinophil activation through binding to CCR3, which in turn promotes pro-angiogenesis and endothelial cell migration, induces the expression of pro-apoptotic genes, and leads to neuronal death, and the concentration of CCL11 in the cerebrospinal fluid is closely associated with the progression of neurodegenerative diseases (Roy-O'Reilly et al., 2017, Liang et al., 2017a, Wang et al., 2017a). It has been shown that the CCL11 gene is associated with the development of lacunar stroke (Liang et al., 2017b).

Ashtin B. Giambrone found a significant increase in CXCL5 in the brains of rat pups by inducing placental ischemia on day 14 of gestation concomitant with cerebral ischemia (Giambrone et al., 2019). This suggests that CXCL5 induces a pro-inflammatory environment in the brains of CCH pups. One study found that CXCL5 expression was increased in the white matter of the brain between 6 and 24 hours after ligating the right common carotid artery and inducing ischemia and hypoxia in the brains of young rats. The damaged microvasculature could recruit activated leukocytes at the damaged white matter through the disrupted blood-brain barrier, leading to persistent neuroinflammation and exacerbating brain injury (Wang et al., 2016a). CCL11 negatively regulates neurogenesis in aged mice under physiological conditions, significantly exacerbates brain injury in adult stroke mice, and promotes neural regeneration in adolescent mice (Lieschke et al., 2019).Meaghan Roy-O 'Reilly found a significant increase in CCL11 levels in young and aged mice 24 hours after experimental stroke, yet found a significant decrease in CCL11 levels in ischemic stroke patients 24 hours after stroke. CCL11 levels at 24 hours after ischemic stroke were significantly associated with in-hospital mortality at patient discharge, and lower CCL11 levels predict stroke severity and can be used as a prognostic marker to predict acute and long-term functional impairment in ischemic stroke patients (Roy-O'Reilly et al., 2017). I/R causes an increase in CXCL5 levels in the cerebrospinal fluid of ischemic stroke patients, which in turn leads to damage of human brain microvascular endothelial cells and blood-brain barrier disruption, and CXCL5 levels are positively correlated with the degree of early cerebral nerve injury (Yu et al., 2021b). Clinical studies have shown that CXCL5 levels are significantly elevated in the serum of CCH neonates (Wang et al., 2016b). Feifei Wang found that CCL11 promotes neural regeneration in neonates with hypoxic-ischemic brain injury by inducing neurogenic precursor cell activation (Wang et al., 2017b). It was found that CXCL5 levels in cerebrospinal fluid of ischemic stroke patients increased rapidly, and CXCL5 levels were positively correlated with the volume of early brain CT hypodense areas (Zaremba et al., 2006). This suggests that CXCL5 may participate in the neuroinflammatory response that accompanies acute ischemic stroke by attracting neutrophils to sites of cerebral ischemia and thus.

CXCL5 induces an aggravated pro-inflammatory environment early in VaD, which can acutely recruit neutrophils and leukocytes at the site of damage, which in turn leads to microvascular dysfunction and disruption of the blood-brain barrier, further aggravating cerebral ischemia. The neurological effects of CCL11 in post-ischemic stroke may be related to the species and age of the subject, and it can be assumed that lower CCL11 levels exacerbate neurological damage.

### CXCL12/SDF-1 and CX3CL1 exert neuroprotective effects in the CI/R and CCH phases of VaD

CXCL12/SDF-1 plays a neuroprotective role in CI/R through the SDF1-CXCR4 axis, which is able to induce neural progenitor cells to nest and migrate to the lesion site (Wang et al., 2022a, Qu et al., 2021, Huo et al., 2022a). sDF-1α is the major isoform of SDF-1. Elevated levels of SDF-1α are a predictor for patients at high risk of transient ischemia (Zhao et al., 2022). The transmembrane anchoring protein CX3CL1 is produced in central neurons and is only expressed in mature neurons at steady state (Subbarayan et al., 2022a). the CX3CL1/CX3CR1 axis maintains crosstalk between neurons and microglia under physiological and pathological conditions and plays a key role in apoptosis. It has been shown that CX3CL1 is neuroprotective and that gene deletion of CX3CL1 increases post-ischemic infarct size and mortality in the brain (Ge et al., 2022). In aging, decreasing CX3CL1 levels can lead to cognitive dysfunction (Subbarayan et al., 2022b).

It has been found that SDF1-α levels in plasma and the number of circulating CXCR4 cells in cerebral ischemic rats over seven days were negatively correlated with 90-day prognostic values (Wang et al., 2022b). Human cord blood endothelial progenitor cells translocated with CXCL12 reduced cerebral atrophy, improved neurobehavioral function, and enhanced neurogenesis and angiogenesis in mice with middle cerebral artery obstruction (Huo et al., 2022b). Xiu-Juan Bai et al. found that increased CX3CL1 expression in rats with middle cerebral artery obstruction reversed pro-inflammatory microglia to anti-inflammatory microglia (Bai et al., 2021a). CX3CL1 and CX3CR1 were able to maintain microglia in a quiescent state, thereby inhibiting the release of inflammatory cytokines. Paulina Pawelec found that injection of exogenous CX3CL1 into the ventricles stimulated endothelial cell proliferation and migration in the ischemic semidark zone, resulting in increased vascular density and a durable neuroprotective and pro angiogenic effect in rodents with cerebral ischemia. In the acute phase after cerebral ischemia, inhibition of CX3CR1 expression exacerbates ischemia-induced chronic cognitive impairment (Pawelec et al., 2020). During cerebral ischemia, neurons release soluble CX3CL1, which reduces neuronal damage by binding to CX3CR1 in brain tissue and inhibiting microglia activation (Bai et al., 2021b).

In the CI/R phase of VaD, CXCL12/SDF-1 promotes neuronal repair and regeneration by recruiting neural progenitor cells to the lesion site, while CX3CL1 reduces neuronal apoptosis by maintaining microglia in a resting state during the CCH phase of VaD. By monitoring the site of high CXCL12/SDF-1 expression after VaD, the site of cerebral ischemia can be localized in real time. The timely maintenance of CX3CR1 expression levels during the CI/R phase may be the direction of research to regulate neural injury during the course of VaD disease.

### Role of CXCL13, XCL-1 and CCL2/MCP-1 in relation to the course of VaD disease

CXCL13 is a potential inducer of CNS autoimmunity, expressed mainly in activated cerebral vasculature, which can inject IL-21-producing T follicular helper cells into the reperfusion phase of brain disease and exacerbate ischemic brain injury.Both XCL-1 and MCP-1 promote apoptosis and exert a tremendous chemotactic effect on THP-1 cells (Yan et al., 2021).XCL-1 exacerbates ischemic brain injury by producing specific chemotactic activity of intellectin protein, which recruits monocytes to sites of inflammation and regulates progenitor cell proliferation. It can promote the clearance of invading bacteria through phagocytosis (Nagata, 2018).CCL2/MCP-1 is produced by a variety of cells, but amoeboid microglia are the main source of CCL2/MCP-1. After cerebral ischemia and hypoxia, amoeboid microglia express CCL2/MCP-1 in large numbers and mediate the migration of microglia along µCP channels, leading to severe neurotoxicity and neurodegeneration (Steiner and Humpel, 2022).

One study found that CXCL13 expression was increased in cerebral ischemic mice exhibiting moderate to severe neurological symptoms, which exacerbated neuronal damage (Guo et al., 2020a).Aditya Rayasam et al. found CXCL13 expression on inflamed cerebrovascular cells and demonstrated that CXCL13 inhibition reduced neuronal death and attenuated secondary brain damage in mice. CXCL13 expression was significantly increased in mice 4 and 24 hours after middle cerebral artery obstruction (Rayasam et al., 2022b).Fengshou Chen et al. found a significant increase in CXCL13 24 hours after ischemia-reperfusion injury in the rat spinal cord. CXCL13 has been shown to be critical to the site of inflammation/injury in neuropathic pain through CXCR5-induced activation of astrocytes, which bind tightly to the CNS microvasculature and thus regulate barrier function (Chen et al., 2020a). However, Katie Z. Chapman et al. found that CXCL13 inhibits apoptosis and promotes neuroblast migration after stroke in rats with middle cerebral artery obstruction, thus suggesting that CXCL13 may be involved in neuroprotection after stroke (Chapman et al., 2015). It has also been found that upregulation of CXCL13 levels in mice with ischemic stroke selectively recruits B cells and attenuates brain injury (Monson et al., 2014). Acidosis is an important cause of irreversible ischemic brain injury. It has been found that after toxic acidosis, the number of activated microglia increased in brain sections of mice with cerebral ischemia, and CCL2/MCP-1 expression was significantly enhanced. Numerous animal studies and clinical trials have shown that CCL2/MCP-1 mediates the pathological process of ischemic stroke and that higher serum levels of CCL2 are strongly associated with a high risk of any form of stroke (Huang et al., 2018, Shan et al., 2021, Bernstein and Rom, 2020). In the CI/R-induced ischemic zone, CCL2 expression is temporal and cell-specific. Knockout of MCP-1 or its receptor in mice with middle cerebral artery obstruction revealed that CCL2 expression was upregulated in their neurons 12 hours after CCH, peaked at 2–3 days, and then gradually decreased. high expression of CCL2 was detected in astrocytes 2 days after CCH. This suggests that CCL2 is expressed in neurons earlier than in astrocytes and that CCL2 plays a role in neurological injury or neuroprotection through CI/R (Geng et al., 2022). It has been found that XCL-1 expression levels were significantly reduced in the cerebrospinal fluid of subjects with post-traumatic brain injury (Habiyaremye et al., 2017). A Meta-analysis showed that patients with the CCL2 rs1024611 gene polymorphism had an increased incidence of ischemic cerebral infarction and that serum CCL2 levels could be used as a biomarker for early detection and prognosis of ischemic cerebral infarction (Geng et al., 2022).CXCL13, XCL-1 and CCL2/MCP-1 are involved in neuroinflammation and exacerbate ischemic brain injury during the CI/R phase of VaD, while exerting neuronal regeneration and neuroprotective effects during the CCH phase. There are fewer studies about the correlation between XCL-1 and VaD, but by observing the changes of CXCL13, XCL-1 and CCL2/MCP-1 expression levels, it may be a useful marker to assess early neurological injury and late prognosis.

## Effects on neuroinflammation in VaD processes after modulation of FCs

**Fig. 1:**
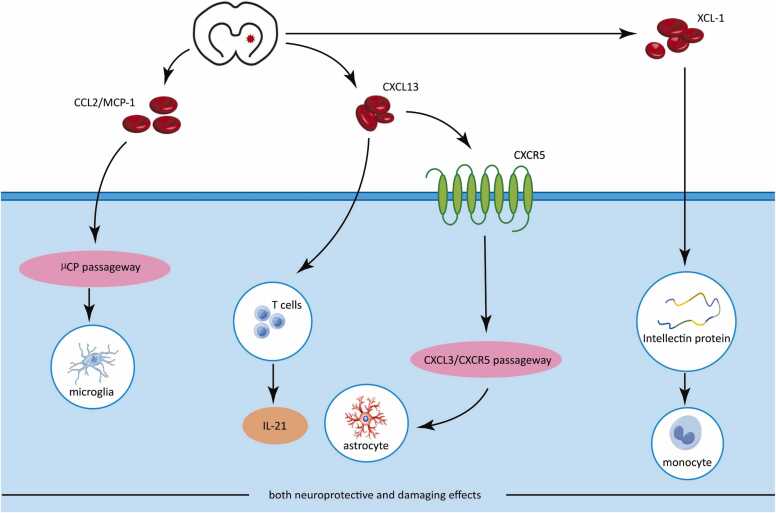
The picture shows the chemokines associated with vascular dementia.The effects of CXCL13, XCL-1 and CCL2/MCP-1 located in the blue area are related to the course of VaD. They participate in neuroinflammation in the CI/R phase of VaD and aggravate ischemic brain injury.

As shown in Fig. 1, FCs levels play an important role in the course of VaD disease. Therefore, modulating the levels of relevant FCs may serve as a potentially effective therapeutic strategy for VaD.

There is growing evidence that neuroinflammation plays a key role in the course of VaD. CCH and thromboembolism in VaD trigger excessive activation of microglia, which can exacerbate neurogenic damage and lead to neurodegeneration and cell death. Recent studies have shown that activated microglia M1 inhibit the inflammatory response by releasing chemokines that trigger neuronal death and exacerbate memory damage. Microglia of the M2 phenotype have anti-inflammatory effects and are involved in the repair of brain injury (Hui et al., 2022). Numerous studies have shown that controlling the polarization of microglia is effective in improving VaD prognosis (Yun et al., 2022, Kang et al., 2022).

Activation of microglia leads to increased secretion of pro-inflammatory cytokines, and damaged microvessels can recruit activated leukocytes at the site of injury, which in turn causes neuroinflammation and disruption of the blood-brain barrier, causing brain aging. Blocking CXCL5 signaling pathway using CXCR2 antagonists significantly attenuates microglia activation and thus reduces neuroinflammation after cerebral ischemia.Lin-Yu Wang et al. showed that mediating SDF-1 expression in astrocytes leads to downregulation of CXCL5 expression and is a viable treatment for sublethal ischemia (Wang et al., 2016c).Chen F suggested that long noncoding RNA SNHG15 is a new target for ischemic stroke therapy by upregulating CXCL13, thereby reducing apoptosis (Guo et al., 2020b). CXCL13 siRNA pretreatment protects rats from spinal cord ischemia-reperfusion injury while decreasing the expression of pro-inflammatory cytokines, and its therapeutic role in CI/R can be further explored at a later stage (Chen et al., 2020b). Novel immunotherapeutic approaches through upregulation of XCL-1 are available, but neurologically relevant studies are yet to be further advanced (Tian et al., 2013). rCX3CL1/CX3CR1 signaling pathway has a large number of clinical applications in the treatment of neurodegenerative diseases (Subbarayan et al., 2022a). ge Y administered exogenous rCX3CL1 to mice with cerebral ischemia-reperfusion and found that rCX3CL1 could inhibit CX3CL1 signaling pathway can be used as a therapeutic target to promote functional recovery after stroke (Ge et al., 2022). Bone marrow stromal cells shift microglia cell type from pro-inflammatory to anti-inflammatory in rats with middle cerebral artery occlusion by secreting CX3CL1, which would provide clues for the treatment of ischemic stroke-related diseases (Bai et al., 2021a).Lieschke S found that intraperitoneal injection of CCL11 significantly exacerbated acute brain injury and significantly impaired post-stroke neurological recovery in adult stroke mice, and administration of CCL11 inhibitors resulted in an increase in microglia and a reversal of these effects. Therefore, interfering with the CCL11 signaling pathway may be an effective approach for future treatment (Lieschke et al., 2019). Bromodopamine-containing protein 4 (BRD4) plays a crucial role in the regulation of inflammation and oxidative stress. dBET1 is a novel and effective BRD4 degrader. Following an ischemic stroke episode, dBET1 administration to mice significantly reduces the expression level of CCL2, which in turn attenuates ischemia-induced microglia and astrocyte-reactive gliosis and improves prognosis (Liu et al., 2022). the CCL2/CCR2 axis in CI/R has been studied accordingly in the clinic, and the development of drugs that modulate the activity of the CCL2/CCR2 signaling pathway can be used to prevent and treat acute phase cellular injury and promote recovery of neurological function in the chronic phase in patients with ischemic stroke (Geng et al., 2022).Extracellular elastin derived peptides (EDPs) accumulate in aging brains and are associated with AD. Scholars have observed that EDPs VGVAPG affect neuronal survival and morphology in a dose-dependent manner, which has led to the development of new targeted therapies for extracellular matrix remodeling in AD (Ma et al., 2024).Glucagon like peptide-1 receptor agonists can restore brain cell homeostasis, regulate microglial activity, and reduce inflammatory responses under inflammatory conditions (Diz-Chaves et al., 2024). There have been studies on the relationship between glucagon like peptide-1 receptor agonists and vascular dementia.Therefore, the use of BRD4 degrading agents such as dBET1, the development of drugs that regulate the activity of the CCL2/CCR2 signaling pathway, the application of EDP targeted therapy, and glucagon like peptide 1 receptor agonists are all future research directions.

Inhibition of CXCL5, CXCL13, XCL-1,and CCL2/MCP-1 during the cerebral ischemia/reperfusion (CI/R) phase of VaD holds promise as a therapeutic strategy to mitigate microglial activation and minimize neurological injury. Conversely, upregulation of CXCL12/SDF-1 or CX3CL1, CXCL13, XCL-1, CCL11, and CCL2/MCP-1 expression during the chronic cerebral hypoperfusion (CCH) phase may provide a viable means of curtailing microglial-mediated neurotoxicity. Some clinical studies have confirmed that chemokines are closely related to vascular dementia (Chen et al., 2021, Zhou et al., 2021).An independent cohort study in Italy showed that a lack of chemokines such as CCR5 can lead to a decrease in neuronal antioxidant stress capacity, thereby increasing the risk of vascular dementia (Tournier et al., 2024). Scholars have found that the levels of CCL2 in the serum of patients over 60 years old with vascular dementia are significantly elevated (Supriya et al., 2023). A prospective study has also confirmed that CCL11 and CXCL9 are closely related to cognitive function in multi-ethnic populations (Elkind et al., 2021).More clinical studies have been conducted using monitoring of single FCs to assess the prognostic risk of patients, but studies using combined monitoring of multiple FCs during the course of VaD and controlling the progression of VaD by modulating the levels of FCs have not been reported, so further studies are needed to investigate the relevance of modulating FCs to VaD.

**Fig. 2:**
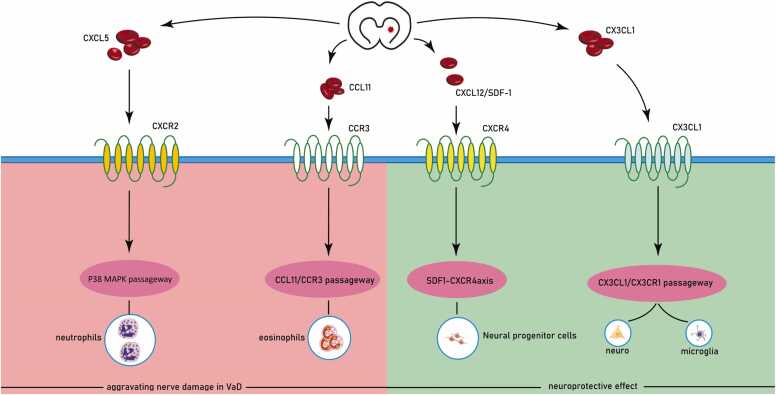
CXCL5 and CCL11 located in the red area can aggravate the nerve damage of VaD. In the CCH phase, it plays a role of neuron regeneration and neuroprotection. CXCL12/SDF-1 and CX3CL1 located in the green region play a neuroprotective role in the CI/R and CCH phases of VaD.

## Prospect

CCH is a key determinant of change throughout the chronic course of VaD, and CI/R can advance the acute progression of VaD. Various FCs have protective or degenerative effects on neuroinflammation during different periods of the VaD disease course. During the CI/R phase, CXCL5 recruits inflammatory cytokines at sites of cerebral ischemia and disrupts cerebral vasculature; activated cerebral vasculature can exacerbate cerebral neuronal injury by expressing CXCL13; meanwhile, XCL-1 and CCL2/MCP-1 promote neuronal apoptosis. CXCL12/SDF-1, which is highly expressed at the site of cerebral ischemia, can promote neural repair and regeneration by recruiting neural progenitor cells. In the CCH phase, prolonged low levels of CCL11 are detrimental to the prognosis of patients with cerebral ischemia; CX3CL1, XCL-1 and CCL2/MCP-1 exert neuroprotective effects. high expression status of CXCL5, CXCL13 and CXCL12/SDF-1 can be used to localize the lesion site. There are few studies on XCL-1 in cerebral ischemia, and the role of XCL-1 in neurological damage during the course of VaD remains to be investigated subsequently. Further studies on the combined monitoring of CXCL5, CXCL13, XCL-1 and CCL2/MCP-1 could be conducted in the future to facilitate early detection of neurological brain injury conditions and assessment of disease progression. Research on targeted therapies for CXCL5 may be a good measure for clinical intervention in the course of VaD. The corresponding modulation of chemokine expression levels during different periods of VaD can contribute to the diagnosis and prognosis of patients. According to current research, it can be found that regulating the expression levels of chemokines at different stages of VaD is helpful for the diagnosis, treatment, and prognosis of patients. Clinically, different chemokine level guidelines can also be developed for patients with different disease courses to control their progression.The activation of microglia is closely related to the progression of neuroinflammation in VaD, and understanding the relationship between changes in FCs levels and microglia can help to improve clinical diagnosis and treatment. Therefore, dynamic monitoring of various related FCs can help to understand and regulate the progression of VaD disease.

## Authors' contributions

(I)Conception and design: JMM

(II) Administrative support: XPY

(III) Provision of study materials: PJF

(IV) Collection and assembly of data: JMM

(V) Data analysis and interpretation:MQZ

(VI) Manuscript writing: JMM

(VII) Final approval of manuscript: All authors

(VIII) The final version has been revised by: ZYC

## Funding

This study was supported partially by the 10.13039/501100001809National Natural Science Foundation of China (81960221 and 82260249 to XPY), the National Science & Technology Fundamental Resource Investigation Program of China (2018FY100903 to XPY), and Jiangxi Provincial Health Commission Science and Technology Plan project (202311506 to ZYC), Jiangxi Provincial Administration of Traditional Chinese Medicine science and Technology Plan project (2022A322 to ZYC).

## CRediT authorship contribution statement

**Manqing Zhang:** Data curation. **Jinming Ma:** Writing – review & editing, Writing – original draft. **Peijie Fu:** Data curation. **Zhiying Chen:** Writing – original draft, Funding acquisition. **Xiaoping Yin:** Funding acquisition.

## Declaration of Competing Interest

The authors report no conflict of interest.
